# TiO_2_/MWCNT/Nafion-Modified Glassy Carbon Electrode as a Sensitive Voltammetric Sensor for the Determination of Hydrogen Peroxide

**DOI:** 10.3390/s23187732

**Published:** 2023-09-07

**Authors:** Rafael Henrique de Oliveira, Daniel A. Gonçalves, Diogo Duarte dos Reis

**Affiliations:** 1 Institute of Physics, Federal University of Mato Grosso do Sul—UFMS, Campo Grande 79070-900, MS, Brazil; rafael.henrique206@gmail.com; 2 Faculty of Exact Sciences and Technology, Federal University of Grande Dourados, Dourados 79804-970, MS, Brazil; daniel.araujogoncalves@gmail.com

**Keywords:** hydrogen peroxide, electrochemical sensor, nanocomposite, cyclic voltammetry, TiO_2_, MWCNTs

## Abstract

In this work we describe a straightforward approach for creating a nanocomposite comprising multiwalled carbon nanotubes (MWCNTs) and titanium dioxide (TiO_2_) using the hydrothermal technique, which is then characterized by scanning electron microscope (SEM), energy-dispersive X-ray spectrometer (EDS), X-ray diffraction analysis (XRD), Fourier transform infrared spectroscopy (FTIR), and thermal gravimetric analysis (TGA) to assess its properties. Nafion is employed as a reticular agent for the nanocomposite on the glassy carbon electrode (GCE), creating the MWCNT/TiO_2_/Nafion/GCE system. The electrochemical behavior of the system was evaluated using cyclic voltammetry, revealing its remarkable electrocatalytic activity for detecting hydrogen peroxide in water. The developed sensor showcased a broad linear response range of 14.00 to 120.00 μM, with a low detection limit of 4.00 μM. This electrochemical sensor provides a simple and highly sensitive method for detecting hydrogen peroxide in aqueous solutions and shows promising potential for various real-world applications, particularly in H_2_O_2_ monitoring.

## 1. Introduction

Hydrogen peroxide (H_2_O_2_) is a simple but significant compound in various applications, such as pharmaceuticals, clinical, environmental, mining, textiles, and food manufacturing. H_2_O_2_ is a signaling molecule regulating essential biological processes, such as immune cell activation, vascular remodeling, and apoptosis. H_2_O_2_ is a secondary product of various enzymatic reactions [1,2,3,4]. Due to its importance as a regulating molecule, hydrogen peroxide is an excellent model molecule for applications in developing new electrochemical sensors.

Electrochemical methods are the most promising for detecting H_2_O_2_ because of their advantages, such as easy miniaturization, rapid response, simple instrumentation, and high specificity and sensitivity. The natural concentration of H_2_O_2_ varies from micromolar (µM) to tens of millimolar (mM) [5]. While various techniques exist for H_2_O_2_ determination, such as chromatography [6], chemiluminescence [7], colorimetry [8], and titrimetric analysis [8], these methods are often complex, expensive, and time consuming. In recent years, many electrochemical approaches have been developed to determine low concentrations of H_2_O_2_ in a high-throughput fashion in fast, simple, reliable, and inexpensive ways [8,9,10].

Electrochemical sensors are suitable for various matrices, require minimal sample preparation, and exhibit high sensitivity and a wide concentration range with low limits of detection [11,12]. Enzymatic and non-enzymatic sensors have been developed for detecting H_2_O_2_ [13,14]. Enzymatic approaches exhibit excellent selectivity and sensitivity but lack stability, require complex and expensive immobilization processes, and are highly dependent on experimental conditions. Therefore, developing non-enzymatic electrochemical H_2_O_2_ sensors is very interesting for various biomedical, industrial, and academic applications. Non-enzymatic electrochemical sensors have been developed by chemically modifying electrodes with nanomaterials, such as nanoparticles of noble metals, transition metals, metallic oxides/hydroxides, bimetallics/alloys, and carbon nanomaterials (CNT, graphene and its compounds) [5,15,16].

Among the promising metals for developing nanomaterials, titanium dioxide stands out. Titanium dioxide (TiO_2_) is a transition metal oxide belonging to the category of n-type semiconductors, with its bandgap energy ranging from 2.9–3.2 eV [17], depending on the crystal phase [18]. Due to its high electrochemical activity, excellent mechanical and chemical stability, and exceptional capacity for organic molecule adsorption, TiO_2_ is widely used in electrochemical sensors [19,20]. However, to further improve the sensing performance of TiO_2_, researchers are working on forming a hybrid structure with different materials with diverse functional properties. One such material extensively studied in electrochemical applications is multi-walled carbon nanotubes (MWCNTs), which possess remarkable physical and chemical properties, such as high electrical conductivity, large surface area, and high energy storage capacity [21]. Previous investigations have shown that modifying electrodes with MWCNTs substantially enhances the rates of electron and proton transfer, resulting in superior peak separation and enhanced electrode sensitivity compared to other types of modified electrodes [22,23]. Additionally, the anchoring of semiconductors’ metal oxide particles onto MWCNTs with homogenous distribution has exhibited improved electronic properties, making it a promising platform for selective sensing and catalytic processes.

Among various nanocomposites, the TiO_2_/MWCNTs nanostructures [24] and other notable composites, such as TiO_2_/MWCNTs/Pt [25,26,27], Au@TiO_2_/MWCNT [28], ZnS/Au10/f-MWCNT [29], and PB-TiO_2_/CNT [30], have been reported to exhibit exceptional electrocatalytic activity toward hydrogen peroxide (H_2_O_2_) and other analytes in biosensing applications. However, it should be mentioned that the growth of complex nanostructures and deposition of catalytic nanoparticles, as previously demonstrated in the literature, typically requires sophisticated techniques such as electrochemical anodization, electrochemical deposition, or photo-induced deposition. Although these techniques have enabled the creation of intricate nanostructures, they can be both time consuming and resource intensive [31].

In this context, the sol–gel technique combined with hydrothermal synthesis has emerged as a promising approach for the preparation of nanometer-sized particles and materials with high specific surface area [32]. TiO_2_/MWCNT nanocomposites can be prepared using this technique, which shows potential for developing chemical and electrochemical sensors or promoting novel applications in biosensing [33] and photodetection [33] thanks to its unique three-dimensional network texture, high surface area, and exceptional electrocatalytic activity.

This study presents a novel sensor for detecting H_2_O_2_ that employs a TiO_2_/MWCNT/Nafion nanocomposite as a surface modifier for a glassy carbon electrode (GCE) in conjunction with cyclic voltammetry (CV). The sensor exhibits high sensitivity and a low detection potential for H_2_O_2_. Notably, this is the first report on the use of TiO_2_/MWCNT/Nafion on GCE for electrochemical H_2_O_2_ detection in water using CV. Our findings indicate that the TiO_2_/MWCNT/Nafion nanocomposite holds promise for developing high-performance H_2_O_2_ sensors with a facile synthesis process, making it a viable candidate for creating sensors for various clinical and environmental applications.

## 2. Materials and Methods

### 2.1. Materials

All chemical reagents used in this study were not subjected to additional purification. Titanium (IV) isopropoxide (TiP, 97.00%) and hydrochloric acid (HCl, 36.00%) were obtained from Sigma-Aldrich (St. Louis, MO, USA). Absolute ethanol (99.5%, CRQ Produtos Químicos, Diadema, Brazil), isopropanol (Sciavicco Comércio e Indústria Ltda, Belo Horizonte, Brazil), and sodium hydroxide micropellets (NaOH, Cromoline Química Fina, Diadema, Brazil). Multi-walled carbon nanotubes (MWCNTs) with an outer diameter of 8–25 nm and length ranging from 5.00 to 30.00 µm and a purity of ≥93% were provided by the Nanomaterials Laboratory of the Department of Physics, UFMG. Nafion-117 was purchased from Sigma-Aldrich (Oakville, ON, USA).

### 2.2. Synthesis of TiO_2_ Particles

**Figure 1:**
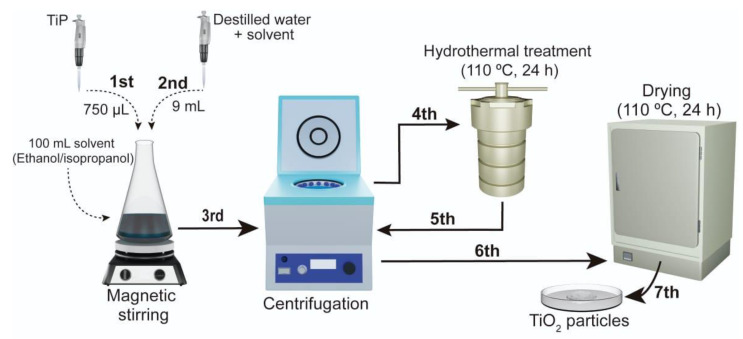
Diagram of preparation of TiO_2_ nanoparticles by combined sol–gel and hydrothermal methods.

The TiO_2_ particles were prepared with modifications by the sol–gel method proposed by Ferreira-Neto, Elias et al. [20], represented in Figure 1. Initially, 750.00 μL of titanium isopropoxide (TiP) was slowly added to 100.00 mL of a mixture composed of ethanol-isopropanol [3:1 (*v*/*v*)] under magnetic stirring, which was maintained for three hours.

Subsequently, 9.00 mL of a mixture of deionized water and solvent (3.00 mL H_2_O:6.00 mL of the ethanol-isopropanol solvent) were added dropwise to form TiO_2_ particles through hydrolysis and condensation of titanium alkoxide species. After 2 h of magnetic stirring, the resulting colloidal suspension was centrifuged at 3500 rpm for 5 min and washed once with the solvent used in the reaction.

To crystallize the amorphous TiO_2_, the obtained material was resuspended in 32.00 mL of deionized water, then transferred to a 35.00 mL hermetic Teflon reactor and subjected to hydrothermal treatment at 110 °C for 24 h inside a flanged stainless steel hydrothermal reactor. After the hydrothermal treatment, the sample was centrifuged and washed twice with deionized water. Finally, the resulting precipitates were dried at 80 °C in an oven for 12 h.

### 2.3. Synthesis of TiO_2_/MWCNT Nanocomposite

**Figure 2:**
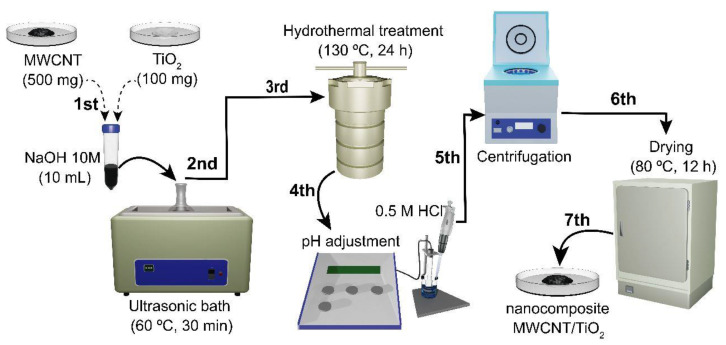
Schematic diagram of the preparation of TiO_2_/MWCNT nanocomposite by hydrothermal method.

The nanocomposite synthesis was adapted from the method Patel B.R. et al. proposed [34]. In this modified method, depicted in Figure 2, 100.00 mg of TiO_2_ particles obtained in the previous step were combined with 50 mg of MWCNTs and added to 10.00 mL of 10 M NaOH solution in a microtube with a screw cap. The resulting mixture was sonicated for 30 min at 60 °C to ensure proper dispersion of the particles. Next, the mixture was transferred to an autoclave container made of stainless steel, lined with Teflon material, and subjected to hydrothermal treatment at 130 °C for 24 h.

After cooling to room temperature, the nanocomposite was washed with 1 L of ultrapure water to remove impurities and unreacted species. The sample was then neutralized to a pH of 7.00 using a 0.50 M HCl solution. To ensure the complete removal of impurities, the nanocomposites were subjected to centrifugation at 5000 rpm with adding 0.50 L of ultrapure water. This washing step was repeated three times.

Finally, the nanocomposite was dried at 80 °C for 12 h to obtain a dry powder. The obtained material was named TiO_2_/MWCNT and was used for further studies.

### 2.4. Materials Characterization

X-ray diffraction analysis (XRD) has been carried out on the Shimadzu diffractometer (model 6100; Kyoto, Japan) using a Co Kα radiation source (λ = 1.788 Å, 40 kV/30 mA). Customary conditions included a 2θ scan from 10° to 70°, a 0.02° angular step, and a 1°/min scan speed. Data were converted to the Cu Kα wavelength using PowDLL software v 2.7 [35]. The crystallographic phase identification was carried out by comparing the obtained patterns with JCPDS standards.

The samples’ chemical structure and surface functional groups were analyzed using a Jasco FT/IR-4100 Fourier transform infrared spectrometer (FTIR) (Tokyo, Japan). The IR spectrum was recorded within the wavelength range of 4000 to 400 cm^−1^.

The structure and surface morphology of the synthesized samples were analyzed using a scanning electron microscope (SEM), model JEOL JSM-6380LV (Tokyo, Japan), equipped with a Thermo Scientific Noran System SIX energy-dispersive X-ray spectrometer (EDS) (Waltham, MA, USA) operated at 20 kV.

Thermogravimetric measurements were conducted using a simultaneous thermal analyzer, the TGA-DSC Netzsch STA 449F3 Jupiter (Selb, Germany), in synthetic air from 30 °C to 900 °C at a heating rate of 10 °C/min.

### 2.5. Sensor Fabrication and Evaluation

A 3.00 mm diameter electrode was polished using 0.05 μm alumina slurry and thoroughly rinsed with distilled water to prepare the glassy carbon electrode for surface modification. A 10.00 μL dispersion of either MWCNTs or TiO_2_/MWCNT nanocomposite in ultrapure water, each at a concentration of 1.00 mg/mL, was then added to the GCE surface. The active materials were immobilized with Nafion^®^–methanol (5.00% *w*/*v*) to create the MWCNT/Nafion/GCE and TiO_2_/MWCNT/Nafion/GCE sensors. One of the unique features of Nafion is its ability to facilitate proton transfer from its sulfonic groups to the perfluorinated hydrophobic backbone, which results in the formation of a highly conductive medium for protons [10,36] to provide greater reticulation of the nanocomposites on the surface of the GCE.

Electrochemical sensing was conducted at room temperature using a three-electrode electrochemical cell (30.00 mL) consisting of the MWCNT/Nafion/GCE or TiO_2_/MWCNT/Nafion/GCE as the working electrode, a Pt plate as the counter electrode, and Ag|AgCl as the reference electrode. The measurements were carried out using a portable bi-potentiostat/galvanostat μ-Autolab Type III (Metrohm Autolab, Utrecht, The Netherlands) controlled by Nova 2.1 software.

The electrochemical sensing was performed in different H_2_O_2_ concentrations in a 0.10 M Britton–Robinson (B-R) buffer at pH 7.00 under constant magnetic stirring. After each triplicate, the solutions were homogenized by stirring for 30 s, and any electrogenerated products were removed from the electrode surface. For the addition–recovery experiments, standard solutions of H_2_O_2_ were prepared using 0.01 M (mol·L^−1^) stock solutions in ultrapure water (R ≥ 18.2 MΩ cm).

## 3. Results

### 3.1. Characterization of MWCNT and TiO_2_/MWCNTs Nanocomposite

**Figure 3:**
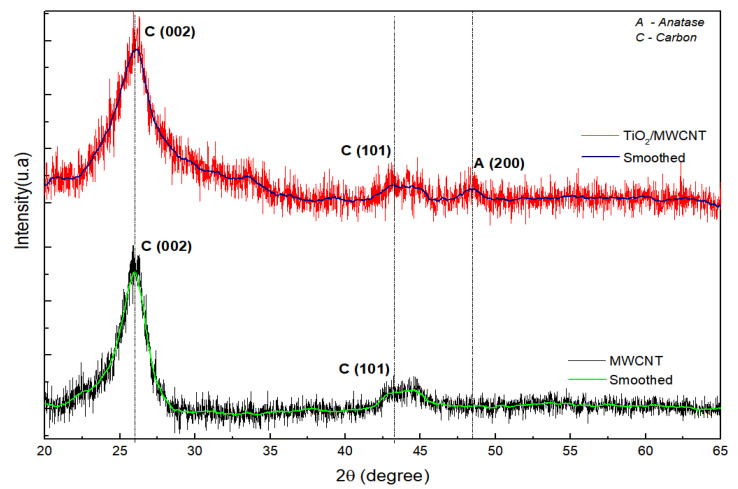
XRD patterns of pristine MWCNT and TiO_2_/MWCNT nanocomposite.

Figure 3 shows the XRD diffraction patterns of both pristine MWCNT and TiO_2_/MWCNT nanocomposite. Although some peaks present low intensity, smoothing has been applied to distinguish them better.

The XRD patterns of pristine MWCNTs show diffraction peaks of graphite structures at 2θ = 25.8° and 43.5° (JCPDS card 41-1487) corresponding to reflections from crystallographic planes (002), the spacing between adjacent graphite layers, and ordering within the plane (100), respectively.

In addition to the diffraction peaks related to pristine MWCNTs, the MWCNT/TiO_2_ nanocomposite exhibits a diffraction peak at 2θ = 48.2° related to the (200) plane, indicating the formation of the anatase phase of TiO_2_ (JCPDS card no. 21-1272). The significant broadening of the peak indicates the nanometric characteristic of the TiO_2_ particles. Thus, the hydrothermal treatment employed was effective, as it promoted the crystallization of amorphous titania into the anatase phase.

An approximate measure of the crystallite size of the TiO_2_ phase in the sample was determined using the well-known Scherrer equation [37], which is based on the full width at half maximum (FWHM) Å obtained by a Gaussian function, diffraction angle θ and the wavelength λ associated with Cu Kα radiation. The average crystallite size of anatase (200) was found to be 5.50 nm based on the broadening of its diffraction peak, where the FWHM is 1.63 Å.

**Figure 4:**
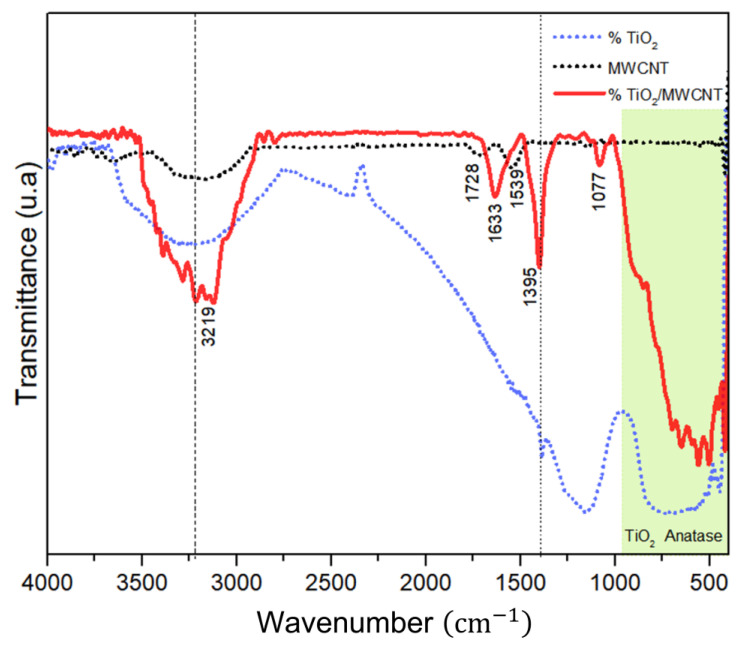
FTIR of MWCNT, TiO_2_ particles, and TiO_2_/MWCNTs nanocomposite.

The FTIR spectra of pristine MWCNTs, bare TiO_2_ particles, and TiO_2_/MWCNT nanocomposite are shown in Figure 4.

The FTIR spectra of all samples showed a broad absorption band in the range of 3000 to 3500 ${cm}^{-1}$, centered at 3219 ${cm}^{-1}$, attributed to the O-H stretching vibration and the surface adsorbed water [38]. In the MWCNTs’ FTIR spectrum, distinct peaks were observed at 1539, 1728, and 3219 ${cm}^{-1}$. The peak at 1539 ${cm}^{-1}$ confirmed the presence of graphitic carbon bonds (C=C stretching vibration), while the peak at 1728 ${cm}^{-1}$ indicated the presence of carbonyl (C=O) functional groups [39,40].

The FTIR spectrum of bare TiO_2_ particles shows a broad band of 1000 to 400 ${cm}^{-1}$, which can be attributed to various stretching vibrations, including Ti–O and O–Ti–O bonds [41]. However, in the FTIR spectrum of the TiO_2_/MWCNT nanocomposite, in addition to the Ti–O and O–Ti–O bonds, the presence of Ti–O–C and Ti–O–C=O bonds is observed, indicating an interaction between TiO_2_ particles and the MWCNTs [42]. Furthermore, the anatase titania phase, as revealed by XRD analysis, contributes to the observed band [43,44]. Both the nanocomposite and bare TiO_2_ particles exhibit a distinct band ~1395 ${cm}^{-1}$ corresponding to TiO_2_ lattice vibrations [45,46]. In addition, the FTIR spectrum of the TiO_2_/MWCNT nanocomposite also displays a peak at 1633 ${cm}^{-1}$, representing the deformative vibration of the Ti–OH stretching mode and the OH stretch of adsorbed water. Moreover, a band at 1100 ${cm}^{-1}$ has been attributed to alkoxy C-O stretching vibrations within the nanocomposite [47].

**Figure 5:**
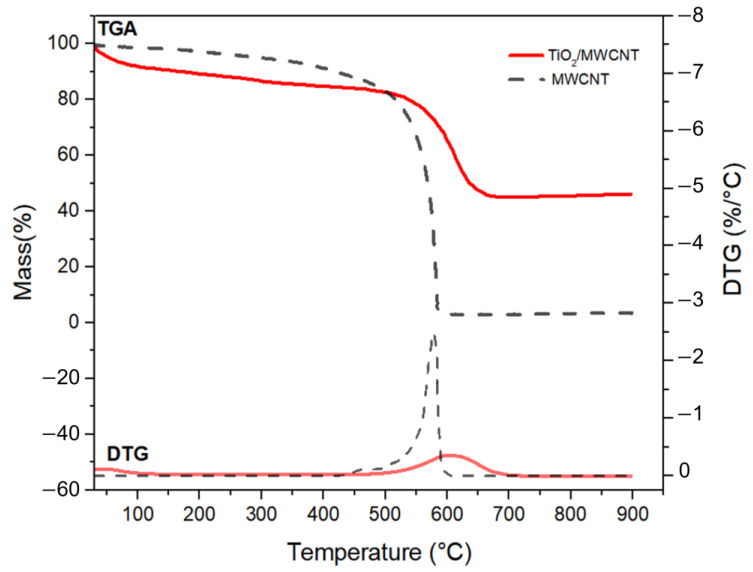
TG and DTG curves for pristine MWCNT and TiO_2_/MWCNT nanocomposite.

Thermogravimetric analysis was used to investigate the thermal stability of the studied materials. According to Figure 5, it can be observed that the MWCNT shows a mass loss caused by the oxidation of the nanotubes with a peak temperature at 575 °C, which agrees with values reported in the literature ranging from 550–650 °C [48]. The stability was achieved with a residue of 3.50%, resulting from the remaining catalysts from the synthesis of the nanotubes (MWCNT), as reported by the supplier of this material.

The MWCNT/TiO_2_ nanocomposite exhibited a mass loss of 5.00% up to 100 °C, which can be attributed to adsorbed water on the material’s surface—this mass loss results from the desorption of the water molecules from the nanocomposite. Between the temperature range of 100 and 450 °C, a further mass loss of approximately 8.00% was observed. This additional mass loss is believed to be caused by the decomposition of oxygenated groups that were incorporated into the MWCNTs during the synthesis process of the nanocomposite [49]. The presence of these oxygenated groups provides evidence for the bonding mechanism between TiO_2_ and MWCNT, suggesting that the bonding occurs through the involvement of these functional groups. This bonding interaction between TiO_2_ and MWCNT is further supported by the presence of Ti–O–C and Ti–O–C=O bonds, as observed in FTIR analysis.

Furthermore, it was observed that the temperature range at which significant mass loss occurs because of the oxidation of nanotubes is between 500–690 °C. This can be easily seen in the DTG curve. The constant levels observed in the TGA curve after 690 °C indicate that the nanotubes underwent complete oxidation. The residue reflects the percentage of TiO_2_ present in the nanocomposite: approximately 41.50%.

**Figure 6:**
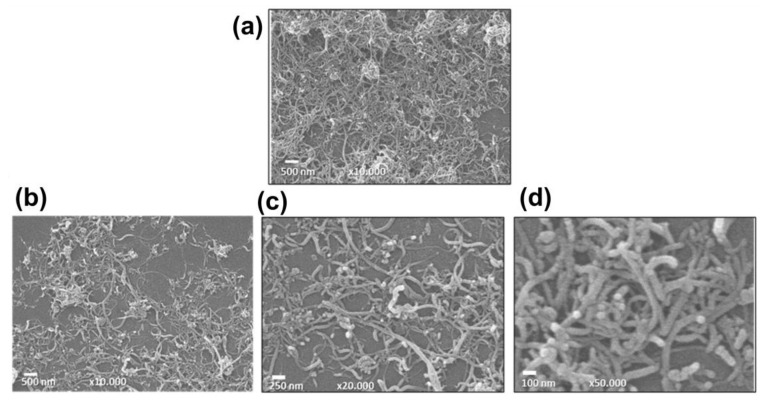
SEM images of pristine MWCNT (**a**) and TiO_2_/MWCNT nanocomposite (**b**–**d**). Scale bars in (**a**,**b**) 500.00 nm, (**c**) 250.00 nm, and (**d**) 100.00 nm.

The morphology of pristine MWCNT and MWCNT/TiO_2_ nanocomposite samples were evaluated using SEM. Figure 6a shows that the pristine MWCNT has a smooth surface with entangled tube bundles. In Figure 6b–d, it can be observed that the MWCNT/TiO_2_ has morphology like the undecorated MWCNTs, i.e., no particles that could be attributed to TiO_2_ were marked.

**Figure 7:**
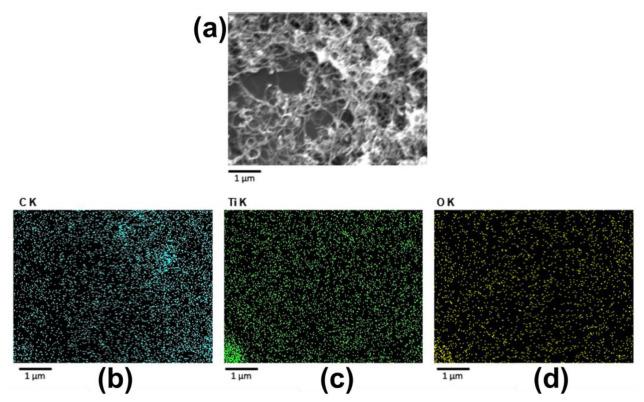
SEM image of the MWCNT/TiO_2_ nanocomposite (**a**) and corresponding EDS elemental mapping with separate maps shown for (**b**) C, (**c**) Ti, and (**d**) O.

The results from the EDS analysis, as shown in Figure 7, support the presence of titanium in the nanocomposite and its successful integration into the carbon nanotube matrix. These findings are consistent with the XRD and FTIR analysis results.

The EDX analysis of the TiO_2_/MWCNT nanocomposite demonstrated its atomic percent (at.%) and weight percent (w.%) composition. The analysis results are as follows: carbon (C)—73.50% (at.%) and 86.72% (w.%), titanium (Ti)—9.22% (at.%) and 8.17% (w.%), and oxygen (O)—17.28% (at.%) and 5.11% (w.%). These findings validate its chemical composition.

### 3.2. Electrochemical Behavior of GCE, MWCNT/Nafion, and TiO_2_/MWCNT/Nafion-Modified GCEs

**Figure 8:**
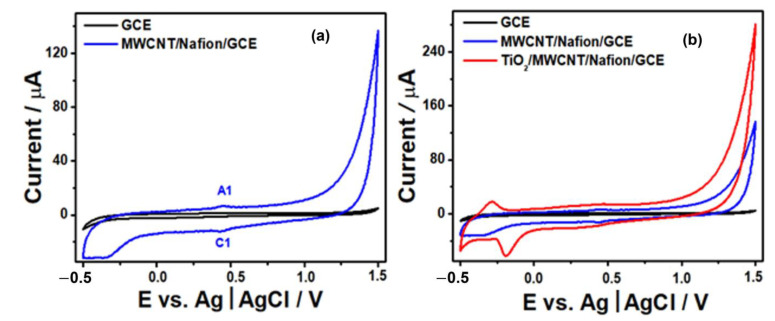
Cyclic voltammograms measured in 0.50 M H_2_SO_4_ within the potential range of −0.5 V to 1.5 V vs. Ag|AgCl at a scan rate of 50 mV$s^{-1}$ using bare GCE, MWCNT/Nafion-modified GCE (**a**), and TiO2/MWCNT/Nafion-modified GCE (**b**).

The electrocatalytic activity of modified working electrodes was examined by measuring the cyclic voltammograms (CVs) in 0.50 M H_2_SO_4_ within the potential range of −0.5 to 1.5 V vs. Ag|AgCl at a scan rate of 50 mVs^−1^. As depicted in Figure 8a, the unmodified GCE was determined to be electrochemically inactive under these conditions, which aligns with the earlier observations reported by Benck et al. [50]. The CV of the bare CGE displayed no redox pair, resulting in a narrow and almost flat line in the graph. This implies that no electrochemical reactions were taking place on the surface of the CGE electrode under the specified conditions.

0.5 V to 1.5 V vs. Ag|AgCl at a scan rate of 50 mV$s^{-1}$ using bare GCE, MWCNT/Nafion-modified GCE (**a**), and TiO_2_/MWCNT/Nafion-modified GCE (**b**).

However, in Figure 8a, the modification of the CGE electrode with MWCNT altered the electrochemical profile of the surface, broadening the area of the voltammogram, as the carbon nanotubes provided a surface and electroactive increment. In addition, two peaks, A1 and C1, are observed, which correlate with the redox reactions of metallic iron resulting from the catalyst used to synthesize MWCNTs [51,52]. Notably, the potential scan toward more negative values for the GCE/MWCNT/TiO_2_ electrode gives rise to redox current peaks in the potential region between −0.5 and 0.0 V, which may be associated with the oxidation/reduction in Ti ions [53,54,55,56].

Moreover, the presence of oxygenated functional groups, such as hydroxyls (OH) and carbonyls (C=O), in the TiO_2_/MWCNT nanocomposite plays a significant role in its electrochemical behavior. These functional groups contribute to increased adsorption capacity for species and provide additional active sites for the adsorption of molecules and ions. Consequently, electrochemical reactions, including the oxidation of H_2_O_2_, can take place more efficiently. This makes the modified GCE electrode an up-and-coming candidate for H_2_O_2_-sensing applications where accurate and sensitive detection of H_2_O_2_ is required [57,58,59].

### 3.3. Evaluation of TiO_2_/MWCNT/Nafion/GCE Electrode as an Electrochemical Sensor for H_2_O_2_ Determination

**Figure 9:**
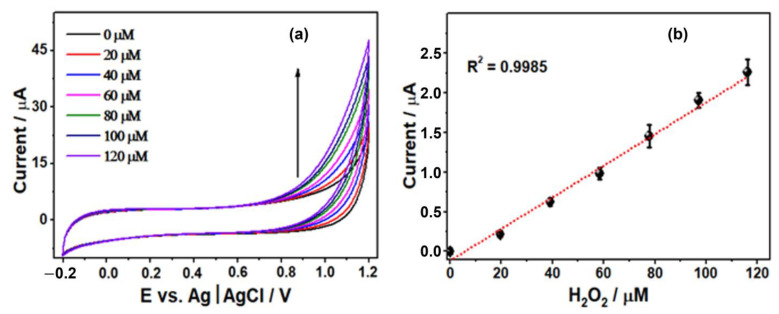
(**a**) Cyclic voltammograms of the TiO_2_/MWCNT/Nafion/GCE to successive addition of H_2_O_2_ concentrations (0.00 up to 120.00 μM) into a continuously stirred B-R buffer solution, pH = 7.00. (**b**) The calibration curve of current vs. H_2_O_2_ concentration.

The electrocatalytic activity of the TiO_2_/MWCNT/Nafion/GCE sensor toward the oxidation of H_2_O_2_ was investigated by CV. Figure 9a shows the cyclic voltammograms of the electrode in a Britton–Robinson (B-R) buffer solution at pH 7, under a potential scan range of −0.2 V to 1.0 V, in the absence of and after successive additions of hydrogen peroxide (from 0 up to 120 μM) to the cell solution, and the corresponding calibration plot.

The cyclic voltammograms demonstrated the direct proportionality of the peak current to H_2_O_2_ concentration within 14.00–120.00 µM. The linear regression equation illustrated Ip (µA) = 1.0145x − 0.0346, (R^2^ = 0.9985) (Figure 9b). The detection limits (LD) and quantification limits (LQ) were calculated according to the recommendations of the IUPAC as three times the standard deviation of the blank signal—Britton–Robinson buffer—(σB) divided by the slope of the calibration curve (m): LD = 3 σB/m. The LQs were calculated similarly, with 10 replacing 3 in each equation, i.e., LQ = 10 σB/m. Therefore, the LD and LQ limits obtained were 4.00 µM and 14.00 µM, respectively.

The fabricated sensor’s accuracy was evaluated using the standard addition method, in distilled deionized water, at three different concentration levels of H_2_O_2_ (20.00, 40.00, and 60.00 µM) Britton–Robinson buffer solution at a pH of 7.00, using cyclic voltammetry.

The results are presented in Table 1, demonstrating a range of variation between 67.30% and 101.90%. Our findings indicate that this analytical method is only sufficiently accurate for concentrations greater than 20.00 µM.

A comparison between the TiO_2_/MWCNT/Nafion/GCE electrode and other GCE-modified electrodes reported in the literature is presented in Table 2. Our results align with those of other studies, indicating that this electrode coating is suitable for detecting H_2_O_2_. Furthermore, it is worth noting that the TiO_2_/MWCNT/Nafion/GCE electrode can be produced with fewer laborious steps than in some of the previous works.

Considering that hydrogen peroxide concentrations typically range from micromolar for in vivo conditions and residual levels in foodstuffs and drinking water to tens of millimolar for bleaching applications and molar for waste treatment applications [67], the TiO_2_/MWCNT/Nafion/GCE has the potential to determine H_2_O_2_ levels in water.

## 4. Conclusions

In summary, a novel H_2_O_2_ voltammetric sensor has been developed by modifying a glassy carbon electrode (GCE) with a TiO_2_/MWCNT/Nafion nanocomposite synthesized using a facile hydrothermal route. Incorporating semiconducting TiO_2_ nanoparticles and highly conducting MWCNTs synergistically enhanced the sensor’s performance toward H_2_O_2_ detection, surpassing the performance of both bare GCE and MWCNT/Nafion-modified GCE electrodes. The proposed modified electrode successfully detected and determined H_2_O_2_ concentrations in water ranging from 14.00–120.00 μM, with a detection limit of 4.00 μM.

These findings represent a significant advancement in the design and fabrication of highly efficient H_2_O_2_-based sensor devices and have important implications for various applications such as water treatment, food safety, and biomedical diagnostics. Using the TiO_2_/MWCNT/Nafion nanocomposite as a sensing material holds great promise for developing cost-effective, reliable, and sensitive H_2_O_2_ sensors with a wide range of detection capabilities.

Further research could explore the potential of this novel sensor platform for other analytes and environmental conditions, paving the way for the development of next-generation sensing technologies. However, it is essential to note that extensive testing and validation of the sensor’s performance under different conditions will be necessary to ensure its practical applicability before commercialization.

## Figures and Tables

**Table 1:** Results for the recovery study in distilled-deionized water at three concentration levels for H_2_O_2_ in Britton–Robinson buffer solution at pH 7.00.

Added (µM)	Found (µM)	Recovery (%)
20.00	13.20 ± 0.11	67.30
40.00	36.02 ± 0.25	92.30
60.00	59.50 ± 0.12	101.90

**Table 2:** Comparison of the proposed sensor with the recently reported H_2_O_2_ sensors.

Modifier Material	Method	LD (µM)	References
MWCNT-POMAF	Amp	0.33	[60]
Co/MWCNT	DPV	1.84	[61]
AuNPs/PSi/Nafion	LSV	14.84	[62]
AuNPs/PSi/Nafion	SWV	15.16	[62]
Hb/MoS	CV	6.70	[63]
Ag/MWCNT	DPV	3.30	[61]
MoS_2_	CV	1.13	[64]
Au@TiO_2_/MWCNT	DPV	1.40	[28]
PB–TiO_2_/fCN	CA	0.088	[30]
TiO_2_/MWCNT	Amp	0.40	[28]
Ni(OH)_2_/ERGO–MWNT	Amp	4.00	[65]
Ag@TiO_2_	CV	0.83	[66]
MWCNT/TiO_2_	CV	4.00	This work

Abbreviations used: Amp (amperometry), DPV (differential pulse voltammetry), LSV (linear sweep voltammetry), SWV (square wave voltammetry), CV (cyclic voltammetry), CA (chronoamperometry), MWCNT-POMAF (multi-walled carbon nanotubes functionalized with polyoxometalate), Co/MWCNT (cobalt/multi-walled carbon nanotubes), AuNPs/PSi/Nafion (gold nanoparticles/porous silicon/Nafion), AuNPs/PSi/Nafion (gold nanoparticles/porous silicon/Nafion), Hb/MoS (hemoglobin/molybdenum sulfide), Ag/MWCNT (silver/multi-walled carbon nanotubes), MoS_2_ (molybdenum disulfide), Au@TiO_2_/MWCNT (gold@titanium dioxide/multi-walled carbon nanotubes), PB-TiO_2_/fCN (phosphate buffer-titanium dioxide/functionalized carbon nitride), TiO_2_/MWCNT (titanium dioxide/multi-walled carbon nanotubes), Ni(OH)_2_/ERGO-MWNT (nickel hydroxide/electrochemically reduced graphene oxide–multi-walled carbon nanotubes), Ag@TiO_2_ (silver@titanium dioxide), MWCNT/TiO_2_ (multi-walled carbon nanotubes/titanium dioxide).

## Data Availability

Not applicable.
